# Association Between Frequency of Childhood Milk Intake and Adult Lung Function

**DOI:** 10.1002/fsn3.72524

**Published:** 2026-10-26

**Authors:** Yu Zhang, Yangyi Zhang, Xiaochi Wang, Chenye Liu, Linyi Ye, Tianxiang Rao, Jingtao Liu, Wenjie Zhang, Yanshuo Li, Ran Wang, Wenfei Bai, Yueqi Li, Yuanmin Yang

**Affiliations:** ^1^ Department of Pharmacy Henan Province Hospital of Traditional Chinese Medicine Zhengzhou China; ^2^ School of Biological Food and Chemistry Shaanxi Xueqian Normal University Xi'an China; ^3^ School of Basic Medical Sciences China Three Gorges University Yichang China; ^4^ Department of Traditional Chinese Medicine Shandong Drug and Food Vocational College Weihai China

**Keywords:** FEV1, FVC, lung function, milk intake

## Abstract

Early‐life nutrition may be associated with subsequent lung function, but evidence regarding milk consumption at different life stages remains limited. We examined the associations between milk consumption frequency during childhood, adolescence, and early adulthood and adult lung function. Data from 3935 adults aged ≥ 20 years in NHANES 2007–2012 were analyzed. Milk consumption frequency was retrospectively assessed for ages 5–12, 13–17, and 18–35 years. Survey‐weighted multivariable linear regression was used to evaluate associations with forced expiratory volume in 1 s (FEV1), forced vital capacity (FVC), and FEV1/FVC, adjusting for relevant covariates. More frequent milk consumption at ages 5–12 years was associated with higher adult FEV1 and FVC. Compared with participants who never consumed milk, those who consumed milk often had higher FEV1 (*β* = 230 mL, 95% CI: 46–414; *p* = 0.016) and FVC (*β* = 324 mL, 95% CI: 120–529; *p* = 0.003) after adjustment. Milk consumption at ages 13–17 and 18–35 years was not significantly associated with FEV1 or FVC. No significant association was observed with FEV1/FVC. More frequent milk consumption at ages 5–12 years was associated with higher FEV1 and FVC in adulthood. These findings suggest that the association between milk consumption and adult lung function may vary according to the timing of exposure. Prospective longitudinal studies are needed to confirm these findings.

## Graphical abstract

This cross‐sectional study of 3935 US adults (NHANES 2007–2012) investigated the link between childhood milk intake and adult lung function. Results indicate that more frequent milk consumption during ages 5–12 years is significantly associated with higher FEV_1_ and FVC in adulthood, independent of smoking status and asthma history. Conversely, milk intake during adolescence (13–17 years) and early adulthood (18–35 years) showed no significant association with lung function parameters. These findings suggest that early‐life nutrition may play a critical role in long‐term respiratory health, highlighting the importance of adequate milk consumption during childhood for optimal lung development.

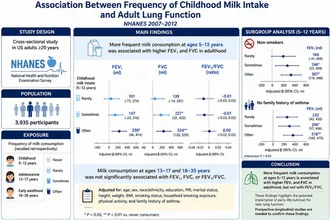

## 1 Introduction

Lung function is an important indicator of respiratory and overall health and is associated with subsequent morbidity and mortality (Duong et al. 2019). Spirometric measures, particularly forced expiratory volume in 1 s (FEV1) and forced vital capacity (FVC), are widely used to characterize pulmonary function and identify airflow limitation and impaired ventilatory capacity (Pellegrino et al. 2005). Although smoking, air pollution, and other environmental exposures are well‐established determinants of adult lung function (Lange et al. 2015; Gauderman et al. 2004), increasing attention has been directed toward potentially modifiable factors earlier in life that may contribute to the attainment and maintenance of optimal lung function (Bui et al. 2018).

Lung growth and maturation extend from the prenatal period through childhood and adolescence into early adulthood (Bui et al. 2018). Nutritional status during these developmental stages may be particularly relevant because adequate intake of energy, protein, vitamins, minerals, and other nutrients contributes to somatic growth and normal physiological development (Arigliani et al. 2018). Previous studies have also suggested associations between dietary factors and respiratory health, potentially involving inflammatory and oxidative pathways (Berthon and Wood 2015; Garcia‐Larsen et al. 2016). However, the role of specific dietary exposures during childhood in relation to lung function later in life remains incompletely understood.

Milk is a commonly consumed food during childhood and provides protein, calcium, phosphorus, and several other micronutrients involved in growth and development (Pereira 2014). Although dairy consumption has been investigated in relation to a range of health outcomes, evidence regarding the association between childhood milk consumption and respiratory health in adulthood remains limited and inconsistent (Berthon and Wood 2015; Bartley and McGlashan 2010). In particular, it remains unclear whether differences in the frequency of milk consumption during childhood are associated with pulmonary function measured later in adulthood. Clarifying this relationship may contribute to a better understanding of the potential links between early‐life dietary patterns and adult respiratory health.

Therefore, using data from the National Health and Nutrition Examination Survey (NHANES), we investigated the associations between milk consumption frequency during childhood, adolescence, and early adulthood and adult lung function, as assessed by FEV1, FVC, and the FEV1/FVC ratio. We further examined whether these associations differed across relevant population subgroups. Given the observational nature of NHANES, the present study was designed to evaluate associations rather than to establish causal or developmental effects.

## 2 Methods

### 2.1 Data Sources

Data were obtained from the National Health and Nutrition Examination Survey (NHANES), a nationally representative cross‐sectional survey of the US non‐institutionalized civilian population. The NHANES protocols were approved by the National Center for Health Statistics Research Ethics Review Board, and written informed consent was obtained from all participants. Because the present study used publicly available deidentified data, no additional institutional ethical approval was required.

### 2.2 Study Participants and Sample Size

Participants with asthma, those aged < 20 years, and those with missing data on milk consumption frequency, spirometric measurements, or smoking information were excluded according to the predefined eligibility criteria (Figure 1).

**FIGURE 1:**
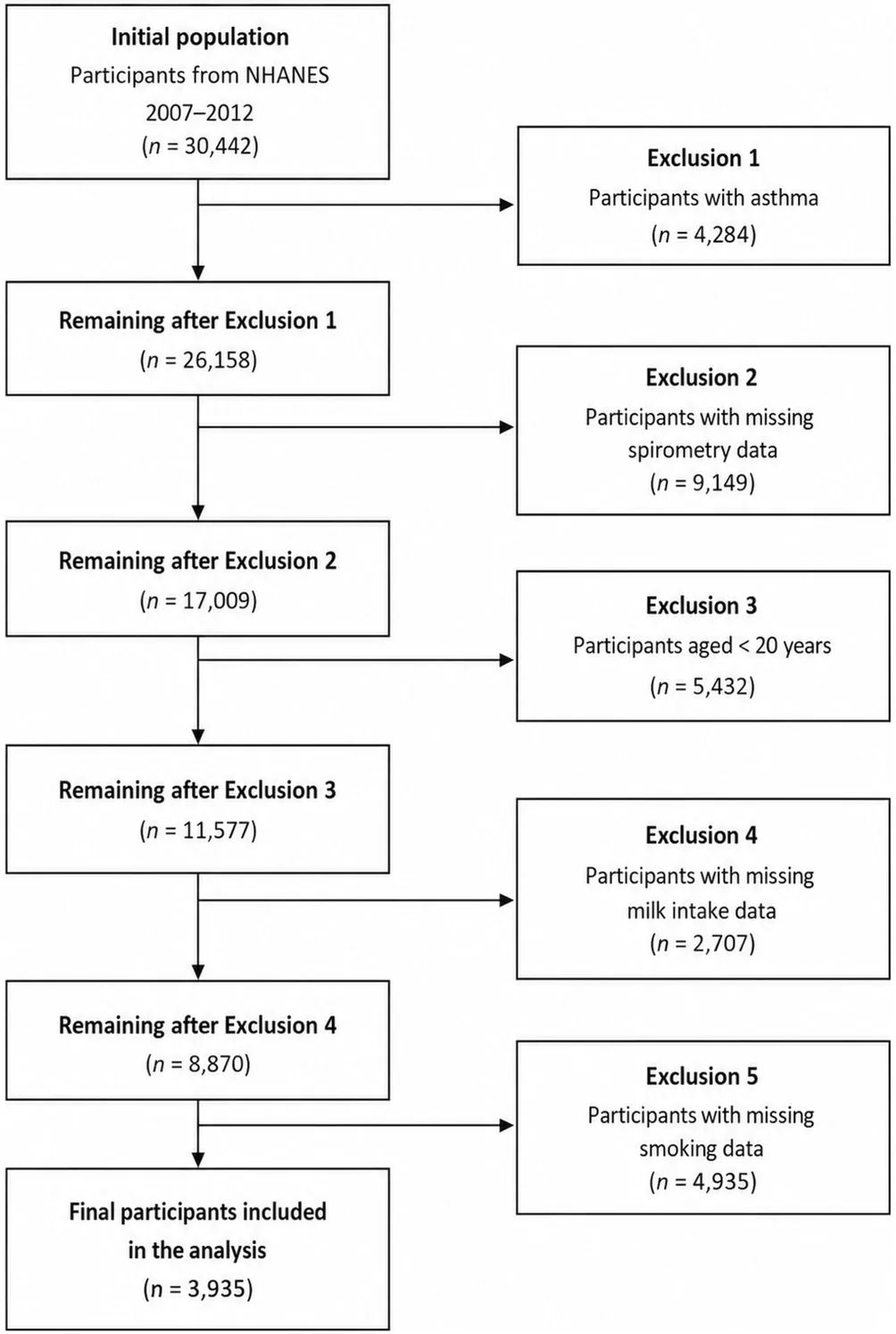
The participant flow diagram.

### 2.3 Assessment of Childhood Milk Intake Frequency

Childhood milk intake frequency was assessed using data from the Dietary Behavior questionnaire in the NHANES. Participants were asked to recall their milk consumption habits during three specific life stages: childhood (5–12 years), adolescence (13–17 years), and early adulthood (18–35 years), corresponding to variables DBQ235A, DBQ235B, and DBQ235C, respectively.

The specific question asked was: “When you were [age range], how often did you drink milk?”

Based on the responses, milk intake frequency was categorized into four ordered levels:

1. Never: Reported drinking milk “Never” (coded as 0).
2. Rarely: Reported drinking milk “Less than once a week” (coded as 1).
3. Sometimes: Reported drinking milk “1 to 3 times a week” or “4 to 6 times a week”.
4. Often: Reported drinking milk “Once a day or more” (coded as 3).

### 2.4 Assessment of Lung Function

Lung function was measured by trained health technicians in the Mobile Examination Center using a dry rolling‐seal spirometer. The quality of each spirometry maneuver was rigorously evaluated based on the American Thoracic Society (ATS) standards.

We evaluated three spirometric outcomes: FEV1, FVC, and the FEV1/FVC ratio. FVC represents the maximum volume of air forcibly exhaled after a maximal inhalation, whereas FEV1 represents the volume exhaled during the first second of the FVC maneuver. The FEV1/FVC ratio was calculated as FEV1 divided by FVC and was used as an indicator of relative airflow limitation.

### 2.5 Covariates

Potential covariates were selected based on prior knowledge of factors potentially associated with milk consumption and lung function. Demographic covariates included age, sex, and race/ethnicity. Race/ethnicity was categorized as Mexican American, Other Hispanic, Non‐Hispanic White, Non‐Hispanic Black, and Other. Socioeconomic covariates included educational attainment (less than 9th grade, 9th–11th grade, high school graduate, some college, and college graduate), marital status (married, widowed, divorced, separated, never married, and living with a partner), and the family income‐to‐poverty ratio (PIR).

Anthropometric measures included weight, height, and body mass index (BMI), with BMI calculated as weight in kilograms divided by height in meters squared (kg/m^2^). Lifestyle‐related variables included smoking status (no smoking, smoking every day, and smoking some days), age at smoking initiation, household exposure to smoking, and participation in vigorous and moderate recreational physical activity. Family history of respiratory disease was represented by whether close relatives had asthma.

FEV1, FVC, and the FEV1/FVC ratio were considered lung function outcomes and were therefore not included as covariates in the corresponding regression models.

### 2.6 Statistical Analyses

All statistical analyses incorporated the NHANES complex survey design, including sampling weights, strata, and primary sampling units, to obtain estimates representative of the US non‐institutionalized civilian population. Continuous variables are presented as means with standard deviations (SDs), and categorical variables as frequencies and percentages. Baseline characteristics were compared across groups using design‐based tests for continuous variables and Pearson's chi‐square tests with Rao–Scott adjustment for categorical variables.

Missing data were assessed for all candidate variables before analysis. Variables with more than 10% missing data were excluded from subsequent analyses. For covariates with ≤ 10% missingness, missing values were imputed using multiple imputation by chained equations (MICE).

Survey‐weighted multivariable linear regression models were used to examine the associations between milk consumption frequency during three age periods (5–12, 13–17, and 18–35 years) and adult lung function. FEV1, FVC, and the FEV1/FVC ratio were analyzed as continuous outcomes, and regression coefficients (*β*) with 95% confidence intervals (CIs) were reported. Participants who reported never consuming milk during each age period served as the reference group. Model 1 was unadjusted. Model 2 was adjusted for age, sex, race/ethnicity, educational attainment, marital status, PIR, height, weight, BMI, smoking status, household smoking exposure, vigorous recreational physical activity, moderate recreational physical activity, and family history of asthma.

To further evaluate overall differences in lung function across milk consumption frequency categories, survey‐design–based overall tests were performed for FEV1, FVC, and the FEV1/FVC ratio. Subgroup analyses were additionally conducted to examine whether the associations between milk consumption at ages 5–12 years and adult lung function differed across selected population characteristics. Subgroup analyses were performed separately for each stratification variable, with mutually exclusive categories defined within each variable. Because each stratification variable was evaluated independently, individual participants could contribute to multiple subgroup analyses across different stratification variables. Effect modification was assessed by including interaction terms between milk consumption frequency and each stratification variable, and the corresponding *p* values for interaction were reported.

All statistical analyses were performed using R software. All tests were two‐sided, and a *p* value < 0.05 was considered statistically significant.

## 3 Result

### 3.1 Participant Characteristics

Table 1 summarizes the baseline characteristics of 3935 participants, representing approximately 52.15 million US adults after application of NHANES survey weights. Participants were stratified by quartiles of the FEV1/FVC ratio. Significant differences across quartiles were observed for age, sex, race/ethnicity, marital status, PIR, weight, height, and BMI (all *p* < 0.05). FEV1 and FEV1/FVC differed significantly across quartiles (both *p* < 0.001), while FVC also showed a significant between‐group difference (*p* = 0.016). Milk consumption frequency at ages 5–12, 13–17, and 18–35 years did not differ significantly across FEV1/FVC quartiles (all *p* > 0.05).

**TABLE 1:** Baseline characteristics of participants stratified by FEV1/FVC quartiles.

Characteristic	*N* ^a^	Overall	FEV1/FVC Q1	FEV1/FVC Q2	FEV1/FVC Q3	FEV1/FVC Q4	*p* ^c^
		*N* = 52,148,8182^b^	*N* = 13,337,3662^b^	*N* = 13,346,9372^b^	*N* = 13,263,4962^b^	*N* = 12,201,0192^b^	
Gender	3935						0.003
Male		2455 (59%)	701 (65%)	619 (60%)	602 (59%)	533 (53%)	
Female		1480 (41%)	283 (35%)	365 (40%)	381 (41%)	451 (47%)	
Age	3935	47 (15)	56 (13)	50 (14)	44 (13)	37 (13)	< 0.001
Race	3935						< 0.001
Mexican American		555 (6.8%)	83 (2.7%)	118 (5.4%)	159 (7.7%)	195 (12%)	
Other Hispanic		369 (4.6%)	59 (1.7%)	83 (3.5%)	104 (5.5%)	123 (8.1%)	
Non‐Hispanic White		1998 (75%)	626 (85%)	528 (78%)	477 (73%)	367 (61%)	
Non‐Hispanic Black		806 (9.6%)	181 (6.6%)	218 (9.8%)	184 (9.3%)	223 (13%)	
Other		207 (4.4%)	35 (3.7%)	37 (3.2%)	59 (4.9%)	76 (6.0%)	
Education level	3935						0.4
Less than 9th grade		385 (5.3%)	100 (4.6%)	100 (6.0%)	95 (5.8%)	90 (4.7%)	
9th–11th grade		743 (15%)	188 (14%)	170 (14%)	168 (13%)	217 (17%)	
High school graduate		1005 (25%)	262 (27%)	249 (24%)	256 (26%)	238 (24%)	
Some college		1133 (33%)	279 (35%)	284 (32%)	281 (32%)	289 (34%)	
College graduate		669 (22%)	155 (20%)	181 (24%)	183 (22%)	150 (20%)	
Marital status	3935						< 0.001
Married		1998 (54%)	552 (62%)	522 (55%)	510 (55%)	414 (44%)	
Widowed		213 (3.5%)	90 (6.6%)	55 (3.4%)	34 (1.9%)	34 (1.9%)	
Divorced		543 (13%)	163 (14%)	152 (16%)	116 (11%)	112 (11%)	
Separated		141 (2.4%)	31 (2.0%)	27 (1.6%)	40 (3.3%)	43 (2.7%)	
Never married		640 (16%)	78 (6.5%)	133 (14%)	174 (17%)	255 (28%)	
Living with partner		400 (11%)	70 (8.6%)	95 (9.9%)	109 (12%)	126 (13%)	
PIR	3935	2.90 (1.62)	3.00 (1.62)	2.93 (1.61)	3.04 (1.62)	2.61 (1.59)	0.003
Weight	3935	83 (20)	82 (19)	83 (20)	85 (20)	82 (21)	0.034
Height	3935	171 (9)	172 (9)	171 (9)	171 (9)	170 (9)	< 0.001
BMI	3935	28.3 (6.2)	27.6 (5.9)	28.2 (5.8)	28.7 (6.2)	28.6 (6.9)	0.015
Milk consumption frequency at ages 5–12 years	3935						0.2
Never		81 (1.6%)	19 (1.3%)	21 (1.3%)	26 (2.2%)	15 (1.6%)	
Rarely		126 (2.2%)	29 (1.8%)	25 (2.4%)	32 (1.8%)	40 (2.9%)	
Sometimes		363 (8.6%)	98 (10%)	82 (6.5%)	90 (8.2%)	93 (9.6%)	
Often		3365 (88%)	838 (87%)	856 (90%)	835 (88%)	836 (86%)	
Milk consumption frequency at ages 13–17 years	3935						0.11
Never		117 (2.8%)	28 (2.3%)	28 (2.9%)	33 (3.3%)	28 (2.8%)	
Rarely		227 (4.9%)	47 (4.1%)	47 (4.3%)	57 (5.0%)	76 (6.5%)	
Sometimes		809 (20%)	174 (18%)	200 (19%)	219 (23%)	216 (22%)	
Often		2782 (72%)	735 (76%)	709 (74%)	674 (68%)	664 (69%)	
Milk consumption frequency at ages 18–35 years	3935						0.082
Never		229 (5.4%)	48 (4.5%)	56 (5.2%)	60 (5.1%)	65 (6.8%)	
Rarely		537 (13%)	118 (12%)	127 (12%)	140 (15%)	152 (14%)	
Sometimes		1263 (33%)	281 (30%)	332 (35%)	325 (33%)	325 (33%)	
Often		1906 (49%)	537 (54%)	469 (48%)	458 (47%)	442 (46%)	
FVC	3935	4352 (1098)	4229 (1185)	4419 (1103)	4441 (1057)	4317 (1021)	0.016
FEV1	3935	3346 (920)	2791 (849)	3333 (828)	3571 (850)	3721 (874)	< 0.001
FEV1/FVC	3935	0.76 (0.09)	0.65 (0.07)	0.75 (0.02)	0.80 (0.01)	0.86 (0.03)	< 0.001
Vigorous recreational activities	3935	742 (22%)	109 (13%)	184 (24%)	212 (26%)	237 (27%)	< 0.001
Moderate recreational activities	3935	1575 (45%)	350 (41%)	396 (46%)	422 (49%)	407 (47%)	0.025
Age started smoking	3935	19 (46)	19 (42)	21 (64)	18 (23)	19 (46)	> 0.9
Smoke	3935						< 0.001
No		1578 (39%)	449 (46%)	394 (38%)	346 (35%)	389 (36%)	
Every day		364 (8.3%)	46 (4.7%)	78 (7.1%)	112 (9.3%)	128 (13%)	
Some days		1993 (53%)	489 (50%)	512 (55%)	525 (56%)	467 (52%)	
Family members smoke	3935	1170 (27%)	347 (33%)	310 (28%)	232 (21%)	281 (25%)	< 0.001
Close relatives have asthma	3935	672 (17%)	155 (16%)	155 (15%)	165 (17%)	197 (19%)	0.3

^a^ *N* not missing (unweighted).

^b^ *n* (unweighted) (%); Mean (SD).

^c^ Pearson's *χ* ^2^: Rao and Scott adjustment; design‐based Kruskal–Wallis test.

### 3.2 The Relationship Between Milk Intake Frequency in Different Periods and Lung Function in Adulthood

Higher frequencies of milk consumption at ages 5–12 years were associated with higher FEV1 and FVC in adulthood, particularly among participants who reported consuming milk sometimes or often (Table 2). Model 1 was unadjusted, whereas Model 2 was adjusted for age, sex, race/ethnicity, educational attainment, marital status, PIR, height, weight, BMI, smoking status, household smoking exposure, vigorous recreational physical activity, moderate recreational physical activity, and family history of asthma. In the fully adjusted Model 2, compared with participants who never consumed milk, those who consumed milk sometimes had higher FEV1 (*β* = 221 mL, 95% CI: 7.3–435; *p* = 0.043) and FVC (*β* = 281 mL, 95% CI: 27–535; *p* = 0.031). Similarly, participants who often consumed milk had higher FEV1 (*β* = 230 mL, 95% CI: 46–414; *p* = 0.016) and FVC (*β* = 324 mL, 95% CI: 120–529; *p* = 0.003). No statistically significant associations were observed among participants who rarely consumed milk. Milk consumption frequency was also not significantly associated with the FEV1/FVC ratio in either model (all *p* > 0.05). Overall, more frequent milk consumption at ages 5–12 years was associated with higher adult FEV1 and FVC, whereas no significant association was observed with the FEV1/FVC ratio. Consistent with the regression findings, the ANOVA results indicated significant overall differences in FEV1 (*F* = 7.21, *p* < 0.001) and FVC (*F* = 8.49, *p* < 0.001) across milk consumption frequency groups at ages 5–12 years. In contrast, no significant between‐group difference was observed for the FEV1/FVC ratio (*F* = 0.47, *p* = 0.703) (Table S1).

**TABLE 2:** Association between milk consumption frequency at ages 5–12 years and adult lung function.

Exposure	Model 1			Model 2		
	Beta	95% CI	*p*	Beta	95% CI	*p*
FEV1						
Never	1 (Ref)			1 (Ref)		
Rarely	74	−296, 443	0.7	−3.7	−227, 220	> 0.9
Sometimes	298	49, 547	0.020	221	7.3, 435	0.043
Often	348	131, 565	0.002	230	46, 414	0.016
FVC						
Never	1 (Ref)			1 (Ref)		
Rarely	97	−350, 543	0.7	29	−249, 307	0.8
Sometimes	339	30, 647	0.032	281	27, 535	0.031
Often	437	178, 696	0.001	324	120, 529	0.003
FEV1/FVC						
Never	1 (Ref)			1 (Ref)		
Rarely	0.00	−0.03, 0.03	0.8	0.00	−0.03, 0.02	0.9
Sometimes	0.00	−0.03, 0.02	0.9	−0.01	−0.03, 0.01	0.4
Often	0.00	−0.02, 0.02	0.7	−0.01	−0.03, 0.01	0.2

In contrast to the findings for milk consumption at ages 5–12 years, no significant associations with adult lung function were observed for milk consumption during the later age periods. Among participants aged 13–17 years, the estimated associations with FEV1 and FVC were relatively modest, and none reached statistical significance across the milk consumption categories (all *p* > 0.05), with all 95% confidence intervals including zero. Similarly, milk consumption at ages 18–35 years was not significantly associated with either FEV1 or FVC, as the effect estimates were small and the corresponding confidence intervals included zero (all *p* > 0.05). Overall, these results suggest that the associations with higher adult FEV1 and FVC observed for milk consumption at ages 5–12 years were not evident for milk consumption at ages 13–17 or 18–35 years (Tables S2 and S3).

### 3.3 Subgroup Analyses

Subgroup analyses identified significant effect modification by age, smoking status, and household smoking exposure for both FEV1 and FVC. For FEV1, significant interactions were observed for age (*p* for interaction = 0.01), smoking status (*p* for interaction = 0.02), and household smoking exposure (*p* for interaction = 0.02) (Figure 2). A significant interaction was also observed for PIR (*p* for interaction = 0.042). The positive association between frequent milk consumption at ages 5–12 years and FEV1 was particularly evident among participants aged 20–44 years (*β* = 248 mL, 95% CI: 39–535; *p* = 0.019), non‐smokers (*β* = 307 mL, 95% CI: 98–516; *p* = 0.005), and those without household smoking exposure (*β* = 384 mL, 95% CI: 180–588; *p* < 0.001). Among participants with PIR > 3.5, frequent milk consumption was also associated with higher FEV1 (*β* = 500 mL, 95% CI: 29–972; *p* = 0.038).

**FIGURE 2:**
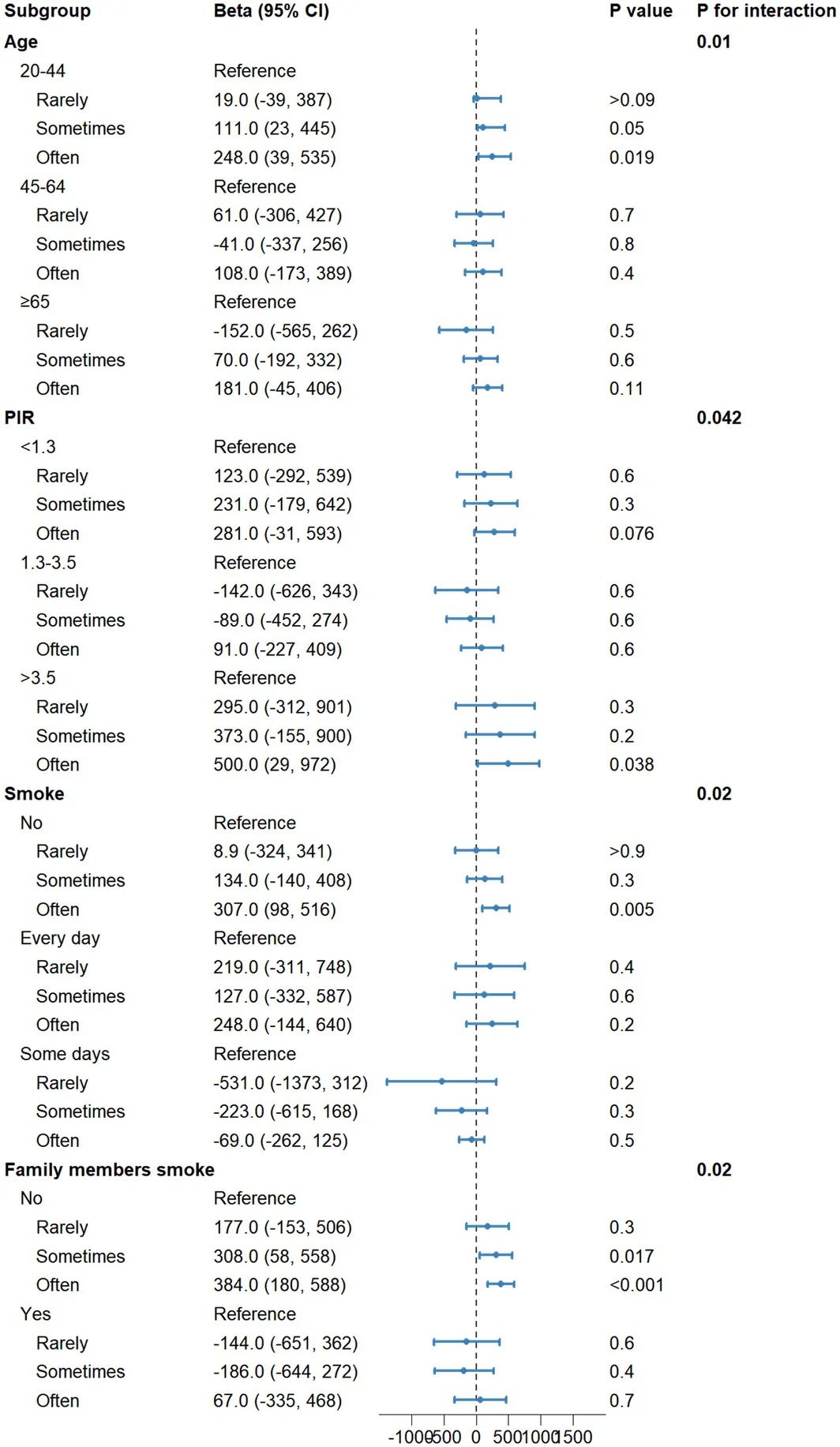
Subgroup analyses of the association between childhood milk consumption frequency (ages 5–12 years) and adult FEV_1_.

For FVC, significant interactions were similarly observed for age (*p* for interaction = 0.01), smoking status (*p* for interaction = 0.03), and household smoking exposure (*p* for interaction = 0.01) (Figure 3). Frequent milk consumption was associated with higher FVC among participants aged 20–44 years (*β* = 265 mL, 95% CI: 93–624; *p* = 0.010) and those aged ≥ 65 years (*β* = 362 mL, 95% CI: 79–645; *p* = 0.013). The association was also evident among non‐smokers (*β* = 495 mL, 95% CI: 275–716; *p* < 0.001) and participants without household smoking exposure (*β* = 537 mL, 95% CI: 306–769; *p* < 0.001). Overall, these findings suggest that the associations of childhood milk consumption with adult FEV1 and FVC varied across selected demographic and smoking‐related subgroups.

**FIGURE 3:**
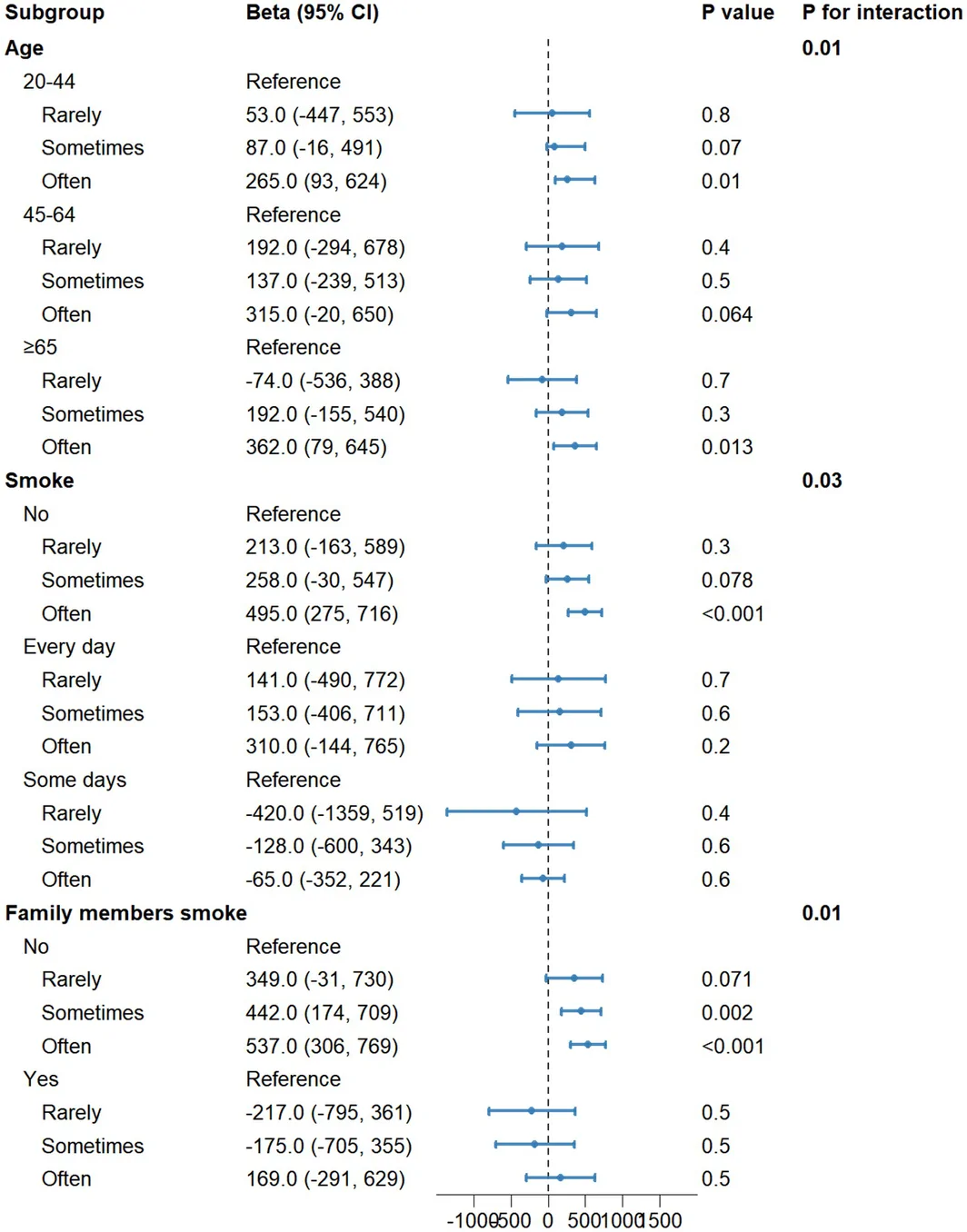
Subgroup analyses of the association between childhood milk consumption frequency (ages 5–12 years) and adult FVC.

## 4 Discussion

In this nationally representative cross‐sectional study using NHANES data, we examined the associations between milk consumption frequency during three life periods (ages 5–12, 13–17, and 18–35 years) and lung function in adulthood. The principal finding was that more frequent milk consumption at ages 5–12 years was associated with higher adult FEV1 and FVC, whereas no significant association was observed with the FEV1/FVC ratio. In the adjusted model, participants who reported consuming milk sometimes or often during ages 5–12 years had significantly higher FEV1 and FVC than those who never consumed milk. In contrast, milk consumption at ages 13–17 and 18–35 years was not significantly associated with adult FEV1 or FVC. Together, these findings indicate that the observed association between milk consumption and adult lung function was most evident for milk consumption reported during childhood rather than during the later life periods examined in this study.

The association observed for milk consumption at ages 5–12 years is consistent with the broader hypothesis that nutritional exposures during childhood may be relevant to subsequent pulmonary function (Jordan and McEvoy 2020; Carraro et al. 2014). Childhood is an important period for overall somatic growth and continued maturation of the respiratory system, during which environmental and nutritional exposures may influence the lung‐function trajectory attained in early adulthood (Jordan and McEvoy 2020; Carraro et al. 2014; Tharumakunarajah et al. 2024). Adequate nutrition during this period may contribute to normal somatic growth and lung development, although the long‐term respiratory effects of individual dietary components remain uncertain (Tharumakunarajah et al. 2024; Dror and Allen 2014). Milk provides protein, calcium, phosphorus, and several other micronutrients involved in normal growth and development (Dror and Allen 2014). These nutritional characteristics provide a possible biological context for the observed associations with FEV1 and FVC; however, the present findings do not demonstrate that the nutrient composition of milk was responsible for these associations (Dror and Allen 2014; Romieu and Trenga 2001). Notably, our findings should not be interpreted as evidence that milk consumption directly promotes lung development or produces lasting structural changes. The observational and retrospective nature of the present study precludes establishing the biological or developmental effects of childhood milk consumption.

An important finding was that similar associations were not evident for milk consumption at ages 13–17 or 18–35 years. This pattern is consistent with, but does not establish, the possibility that the timing of dietary exposure may be relevant to its relationship with later lung function. Lung‐function trajectories evolve across childhood and adolescence and may not reach their maximum levels until early adulthood (Karmaus et al. 2019). Nutritional requirements, growth rates, dietary patterns, and other environmental exposures also vary considerably across childhood, adolescence, and adulthood, and these differences may contribute to the age‐specific patterns observed in the present analysis. Alternatively, the absence of statistically significant associations during the later periods may reflect differences in exposure distribution, recall accuracy, residual confounding, or statistical power. In particular, retrospective assessment of dietary intake many years after the exposure period is susceptible to measurement error and may be influenced by participants' current dietary habits (Dwyer et al. 1989; Chavarro et al. 2009). Therefore, the present findings should not be interpreted as demonstrating that milk consumption after age 12 has no relevance to respiratory health. Longitudinal studies with prospectively collected dietary information and repeated lung‐function measurements are needed to determine whether childhood represents a particularly important period for the association between milk consumption and later pulmonary function (Karmaus et al. 2019).

The subgroup analyses further suggested that the association between childhood milk consumption and adult lung function was heterogeneous across selected participant characteristics. Significant interactions were observed for age, smoking status, and household smoking exposure for both FEV1 and FVC, while PIR also showed evidence of interaction for FEV1. In particular, frequent childhood milk consumption was associated with higher FEV1 and FVC among participants aged 20–44 years and among non‐smokers. Associations were also more evident among participants without household smoking exposure. For FEV1, an association with frequent milk consumption was additionally observed among participants with PIR > 3.5. These findings suggest that demographic, socioeconomic, and smoking‐related characteristics may modify the observed association between childhood milk consumption and adult lung function. Nevertheless, subgroup findings should be interpreted cautiously because multiple comparisons were performed, some subgroup estimates had relatively wide confidence intervals, and subgroup analyses are particularly susceptible to chance findings when numerous comparisons are conducted (Wang et al. 2007; Brookes et al. 2004). Moreover, statistical significance in one subgroup but not in another does not by itself establish a true difference between subgroups (Wang et al. 2007; Brookes et al. 2004). Accordingly, greater emphasis should be placed on formal interaction tests rather than on isolated subgroup‐specific *p* values.

Several mechanisms could potentially contribute to the observed association, although these mechanisms were not directly evaluated in the present study. Milk contains high‐quality protein, calcium, phosphorus, and other nutrients involved in childhood growth and tissue development (Thorning et al. 2016). Milk consumption has also been associated with circulating insulin‐like growth factor 1 (IGF‐1) concentrations in children, although the clinical and respiratory implications of this association remain uncertain (Hoppe et al. 2004). Because body size—particularly height—and pulmonary volumes are closely related, nutritional status during growth may be associated with the attainment of adult lung volumes (Quanjer et al. 2012). In addition, dietary factors may influence inflammatory and oxidative processes relevant to respiratory health (Schünemann et al. 2001). However, milk consumption frequency in NHANES does not provide direct information on individual nutrient intake, biological pathways, or structural lung development. Therefore, explanations involving specific pathways, such as IGF‐1 signaling, antioxidant protection, or immune modulation, remain speculative in the context of our findings and require confirmation in mechanistic or prospective studies.

The present findings may have implications for understanding the relationship between early‐life dietary patterns and adult respiratory health. Previous research on lung function has primarily emphasized established determinants such as smoking, environmental exposures, respiratory disease, and socioeconomic conditions (Eisner et al. 2010; Hegewald and Crapo 2007), whereas the potential contribution of childhood dietary exposures has received comparatively less attention. Our results add population‐based evidence of an association between childhood milk consumption frequency and adult FEV1 and FVC. Importantly, the absence of a significant association with the FEV1/FVC ratio suggests that the observed pattern may be more closely related to differences in absolute lung volumes than to airflow limitation. However, the present results do not establish that increasing childhood milk consumption would improve adult lung function or prevent respiratory disease. Further prospective studies are needed to determine whether the observed associations are reproducible and to clarify the potential roles of overall childhood diet, growth, and related socioeconomic and environmental factors.

This study has several strengths, including the use of data from NHANES, a nationally representative survey employing a complex, multistage probability sampling design (Johnson et al. 2013); standardized spirometric measurements (Graham et al. 2019); and consideration of a broad range of demographic, socioeconomic, anthropometric, lifestyle, and family‐history factors. The use of survey‐weighted analyses also allowed the complex sampling design of NHANES to be taken into account (Johnson et al. 2013). Furthermore, evaluating milk consumption across different age periods provided an opportunity to examine whether associations with adult lung function differed according to the reported timing of exposure.

Several limitations should also be considered. First, because of the cross‐sectional and retrospective nature of the analysis, the temporal sequence between the exposure and outcome cannot be definitively established, and causal relationships cannot be inferred (Wang and Cheng 2020). Although milk consumption was reported for earlier life periods and lung function was measured in adulthood, childhood milk consumption was recalled retrospectively by adult participants, making the exposure assessment susceptible to recall error and exposure misclassification. Measurement error is a recognized challenge in nutritional epidemiology and can bias estimated diet–disease associations (Freedman et al. 2011). Second, milk consumption was assessed according to frequency rather than quantity, type of milk, or nutrient intake; therefore, the observed associations cannot be attributed to specific components of milk. Third, residual and unmeasured confounding remains possible despite adjustment for multiple covariates (Fewell et al. 2007). Childhood milk consumption may be associated with childhood socioeconomic circumstances, overall dietary quality, growth patterns, physical activity, and other health‐related behaviors that were not fully captured in the available data. Fourth, a substantial proportion of potentially eligible participants were excluded because of missing milk‐consumption, spirometric, or smoking information. If the probability of having complete data was related to both milk consumption and lung function, these exclusions may have introduced selection bias (Hernán et al. 2004). Finally, the subgroup analyses involved multiple comparisons, and some strata contained relatively small numbers of participants, resulting in imprecise estimates and increasing the possibility of chance findings (Sun et al. 2012). These subgroup findings should therefore be regarded as exploratory and require confirmation in independent populations.

Overall, more frequent milk consumption at ages 5–12 years was associated with higher FEV1 and FVC in adulthood, whereas comparable associations were not observed for milk consumption at ages 13–17 or 18–35 years. No significant association was identified with the FEV1/FVC ratio. These findings are consistent with a potential relationship between childhood dietary patterns and adult lung function, but they do not establish a causal or structural developmental effect of milk consumption. Prospective longitudinal studies incorporating detailed dietary assessment and repeated measurements of growth and pulmonary function are needed to clarify the temporal and biological basis of these associations.

## 5 Conclusion

In conclusion, more frequent milk consumption at ages 5–12 years was associated with higher FEV1 and FVC in adulthood, whereas no significant association was observed with the FEV1/FVC ratio. Comparable associations were not evident for milk consumption at ages 13–17 or 18–35 years, suggesting that the observed relationship may vary according to the timing of milk consumption. These findings provide epidemiological evidence for a potential association between childhood dietary habits and adult lung function. However, given the observational and retrospective nature of the study, causal or developmental effects cannot be established. Further prospective longitudinal studies are warranted to confirm these findings and clarify the potential role of childhood nutrition in adult respiratory health.

## Author Contributions

**Yu Zhang:** writing – review and editing. **Yangyi Zhang:** writing – original draft, funding acquisition. **Xiaochi Wang:** methodology, formal analysis. **Chenye Liu:** visualization, validation, writing – review and editing. **Linyi Ye:** validation, formal analysis, project administration. **Tianxiang Rao:** investigation, visualization. **Jingtao Liu:** validation, funding acquisition. **Wenjie Zhang:** methodology, project administration, writing – review and editing. **Yanshuo Li:** methodology, validation, writing – review and editing. **Ran Wang:** conceptualization, writing – original draft. **Wenfei Bai:** funding acquisition, visualization, formal analysis. **Yueqi Li:** writing – review and editing, funding acquisition. **Yuanmin Yang:** writing – review and editing, funding acquisition.

## Funding

This work was supported by the 2025 Special Research Project in Pediatrics and Pharmacy (Grant No. GWJJZX20251007089); the Natural Science Foundation of Henan Province (Grant No. 252300423824); the Joint Construction Project of Traditional Chinese Medicine Research in Henan Province (Grant No. 2025LHZX2004); and the Doctoral Research Start‐up Fund Project of Henan Provincial Hospital of Traditional Chinese Medicine (GrantNo.2024BSJJ04).

## Conflicts of Interest

The authors declare no conflicts of interest.

## Supporting information


**Table S1:** ANOVA of lung function across childhood milk intake frequency groups.
**Table S2:** Association between milk consumption frequency at ages 13–17 years and adult lung function.
**Table S3:** Association between milk consumption frequency at ages 18–35 years and adult lung function.

## Data Availability

Publicly available datasets were analyzed in this study. These data can be found at: www.cdc.gov/nchs/nhanes/.
