# Supplementary material for: Association Between Frequency of Childhood Milk Intake and Adult Lung Function
Source: Food Sci Nutr. 2026 Oct 26;14(11):e72524. doi: 10.1002/fsn3.72524 (PMC13710669; doi:10.1002/fsn3.72524)
Supplement: Supplementary file 1 [file FSN3-14-e72524-s001.docx]

| **Supplementary Table.1** ANOVA of Lung Function Across Childhood Milk Intake Frequency Groups | | | | | |
| --- | --- | --- | --- | --- | --- |
| Outcome | term | F_statistic | df1 | df2 | p.value |
| FEV1 | X5_12_factor | 7.209811926 | 3 | 46 | 0.000458338 |
| FVC | X5_12_factor | 8.494447603 | 3 | 46 | 0.000134449 |
| FEV1/FVC | X5_12_factor | 0.471698558 | 3 | 46 | 0.703486983 |

| **Supplementary Table.2 Association Between Milk Consumption Frequency at Ages 13–17 Years and Adult Lung Function** | | | |
| --- | --- | --- | --- |
| **Exposure** | **Model1** |  |  |
|  | **Beta** | **95%CI** | **P value** |
| **FEV1** |  |  |  |
| Never | 1 (Ref) | - | - |
| Rarely | 35 | -85 - 155 | 0.56 |
| Sometimes | 62 | -45 - 169 | 0.25 |
| Often | 88 | -30 - 206 | 0.14 |
| **FVC** |  |  |  |
| Never | 1 (Ref) | - | - |
| Rarely | 45 | -110 - 200 | 0.56 |
| Sometimes | 75 | -60 - 210 | 0.27 |
| Often | 95 | -55 - 245 | 0.21 |
| **FEV1/FVC** |  |  |  |
| Never | 1(Ref) |  |  |
| Rarely | 0.00 | -0.03, 0.03 | 0.89 |
| Sometimes | 0.00 | -0.02, 0.02 | 0.70 |
| Often | 0.00 | -0.02, 0.03 | 0.86 |

| **Supplementary Table.3 Association Between Milk Consumption Frequency at Ages 18–35 Years and Adult Lung Function** | | | |
| --- | --- | --- | --- |
| Exposure | Model1 |  |  |
|  | Beta | 95%CI | P value |
| FEV1 |  |  |  |
| Never | 1 (Ref) | - | - |
| Rarely | 12 | -120 - 144 | 0.85 |
| Sometimes | -15 | -135 - 105 | 0.80 |
| Often | 25 | -110 - 160 | 0.71 |
| FVC |  |  |  |
| Never | 1 (Ref) | - | - |
| Rarely | 20 | -180 - 220 | 0.84 |
| Sometimes | -10 | -200 - 180 | 0.91 |
| Often | 38 | -150 - 226 | 0.69 |
| **FEV1/FVC** |  |  |  |
| Never | 1(Ref) |  |  |
| Rarely | 0.00 | -0.03, 0.03 | 0.82 |
| Sometimes | 0.00 | -0.03, 0.04 | 0.84 |
| Often | 0.00 | -0.03, 0.02 | 0.90 |
